# Effect of maternal zinc supplementation on offsprings hatchability, growth performance and intestinal development in broiler chickens

**DOI:** 10.1038/s41598-025-15502-x

**Published:** 2025-10-14

**Authors:** Marappan Gopi, Govinthasamy Prabakar, Jaydip Jaywant Rokade, Gautham Kolluri, Beulah V. Pearlin, Jagbir Singh Tyagi, Jag Mohan

**Affiliations:** 1 ICAR - Central Avian Research Institute, Izatnagar, 243 122 India; 2 ICAR - National Institute of Animal Nutrition and Physiology, Bengaluru, 560030 India

**Keywords:** Broiler breeders, Zinc-methionine, Progeny performance, Hatchability, Intestinal morphometry, Cell growth, Embryogenesis

## Abstract

Maternal diet plays a vital role in the development of the offspring and their post-hatch performance. The present study was conducted to evaluate the effect of supplementary organic zinc to the basal diet of broiler breeder chickens on the bird’s fertility, hatchability, production performance of the progenies and their intestinal morphological attributes. Broiler breeders (128) were grouped into four as control, fed basal diet containing 40 mg inorganic Zn/kg feed and groups of T1, T2 and T3 fed control diet with 20, 40 and 60 mg/kg organic Zn respectively, fed according to the breeder’s nutrient specifications except zinc content. The organic zinc was supplemented as zinc-methionine. Group-wise fertility and hatchability of the breeder birds were determined. The group-wise hatched chicks were reared for 42 days to assess their production performance, intestinal length and histo-morphology including villi length, diameter, crypt depth and corresponding absorptive surface. The hatchability was higher in 40 and 60 mg/kg supplemented groups. The hatch weight (g) and post-hatch growth performance of chicks were similar (*P* > 0.05) among the groups and were not influenced by the level of organic zinc supplementation. However, the intestinal histo-morphological attributes (villi length, diameter and mucosal cells) showed significant (*P* < 0.05) improvement in chicks hatched from the breeder’s fed with organic Zn supplemented (60 mg/kg of Zn) birds followed by 20, 40 mg and control group. The results indicated that the maternal zinc supplementation had positive impact on breeder’s fertility, hatchability, and intestinal histo-morphological attributes of the offspring but not on the performance of the offspring’s.

## Introduction

Global population is expected to grow by 18.52% to reach 9.6 billion in 2050 from the 8.5 billion in 2024^1^. This will directly increase the requirement for animal protein sources. Among the food animals sector, poultry is one of the fastest growing industry along with the aquaculture. The poultry sector is dominated by broilers and layers due to their affordability and acceptance. Recently, significant advances have been achieved in broiler birds due to their short lifespan. With improvements in breeding/selection techniques, precise nutrition and management, today’s broiler chicken are able to attain a market weight of 2.2 kg within a span of 35 days from their initial hatch weight of 40-45 g chick with a daily 63.1 g gain per day^2^. This level of performance corresponds to a growth rate of 55 times of chick weight in a very short period.

Unlike mammals, the chick spend almost 40% of their lifespan (21 days) inside the egg, from the time the fertile eggs are laid. Considering the significant amount of time spent in the hatchery and the physiological nature of eggs to handling, it allows interventions for better production performance, immunity, health and welfare. The technique of *in ovo* administration of nutrients such as minerals, vitamins, amino acids^3^, non-nutrients like osmo-regulators, pro/prebiotics, and even vaccinations are being performed to improve post-hatch performance, health and welfare of chicks^4^. Although the *in ovo* method has much advantages, it is expensive and could reduce the hatchability^5^.

Nutrients required for the embryo to develop during the incubation, relies on the availability within the egg when it is laid. The nutrients in the parental diet will dictate the growth, development, nutrient metabolism, health and physiological status of the progeny^6^. In addition to offspring’s performance, the manipulation of breeder’s diet could influence the fertility and hatchability percent of the eggs increasing the production of broiler chicks^7^. Among the macro and micro nutrients fed to the breeders, macro nutrients like protein, energy and amino acids could improve the breeder’s production performance (body weight gain, feed consumption and feed efficiency)^8^. However, fortifying the breeder’s diet with micro nutrients such as the minerals and/or vitamins could directly impact the fertility and hatchability of the eggs, embryo and progeny development^9^.

Among the various micro-nutrients, zinc (Zn) is one of the important mineral for breeder birds (both male and females), as it is involved in various biological functions like transforming the synthesis of protein, carbohydrate and nucleic acid, activating the enzymes, spermatogenesis and anti-oxidant defense mechanism which is essential for the proper functioning of the immune system^10,11^. Zn also act as the center point in various cellular metabolism and is involved in cell healing, proliferation and differentiation functions through both structural as well as catalytic effects in biochemical pathways of enzyme systems, DNA synthesis, and gene expression^12,13^. Incorporation of Zn to the broiler breeder diet improves the fertility and is also helpful in bone and feather development in them, and scavenging the oxygen free radicals^14,15^.

Arbitrarily increasing the inorganic Zn content in the breeder diet, could lead to enteric mineral–mineral antagonism (among bivalent cations) and higher mineral excretion leading to environmental concerns^16^. However, the use of organic Zn has been reported to have higher bioavailability than the inorganic sources and avoids mineral antagonism in the gut^17^. Considering the importance of parental diet and requirement to essentiality to reduce the mineral excretion, the present study investigated the effect of supplementary organic zinc (Zn-methionine) to broiler breeders, during the post peak phase, on their egg production, egg weight, fertility, hatchability, chick weight, post-hatch performance and gut development in the offspring.

## Materials and methods

### Ethical approval

The experimental protocols used in the experiment were approved by the Institute Animal Ethics Committee (IAEC) and Committee for Control and Supervision of Experiments on Animals (CCSEA). The IAEC and CCSEA approval number for the study is: CARI/IAEC/ab/4572. The study was carried out in accordance with the ARRIVE guidelines with respect to the study design, experimental animals and numbers, randomization procedure and statistical analysis, etc.

### Experimental birds

The experiment was carried out in the institute’s broiler breeder unit. A total of 128 adult healthy white broiler breeders (CARIBRO Vishal) of 40 weeks age from same hatch were used for this developmental study. The birds were randomly assigned into four groups each comprised of eight males and twenty-four females in eight replicates. The male birds were maintained in individual cages while the females were reared in colony cages of three with access to pre-weighed feed and water. The males and females were reared separately in open sided ventilated sheds for twelve weeks. The initial body weight of male and female breeder birds were 3910 and 3125 g, respectively. The body weight of both the males and female birds among the groups were similar (*P* > 0.05) within the respective sex. The body weight of birds were recorded at the start as well as at the end of the feeding trial. The feed consumption at the start and end of the experiment were also recorded. No mortality among the groups were observed during the experiment.

### Diet formulation

The male and females were fed with separate breeder’s diet (mash feed) throughout the experimental duration. The diets consisted of basal ration (control group, contained 40 mg zinc/kg of feed), basal diet supplemented with 20 (T_1_), 40 (T_2_) and 60 mg of Zn/kg of feed (T_3_). The additional supplementary zinc was incorporated as organic Zn in the form of Zn-methionine (16% elemental Zn). The total Zn content in the groups were 40, 60, 80 and 100 mg/kg of feed. The male and female ration consisted 14 and 18% crude protein content, respectively as per the recommendations of ICAR^18^.

### Egg production, semen collection and insemination

The replicate-wise egg weight and the egg production were recorded daily to derive the hen-day egg production % (HDEP) using the formula:

$$Hen\,day\,egg\,production \,(\% ) = \frac{Total\,number\,of\, eggs\, laid \,on \,a \,given\, day}{{Total \,number \,of\, birds \,on\, that\, day}} \times 100$$

The semen collection was performed by the abdominal massage method^19^. The birds were held by the thighs in crouching position and were gently massaged in the lumbar region for 3 to 4 times starting from head side to the tail with palm. This repeated process stimulated the copulatory organ to protrude out from the cloacal region. Using the thumb and forefinger of the same massage hand, the copulatory organs was gently squeezed to discharge the semen (white, thick and creamy), which were collected in wide mouth glass funnel. The group-wise collected semen were pooled and diluted with CARI Poultry Semen diluent (patented) in 1:2 ratio. Hens were inseminated immediately with diluted semen (1:2) through vaginal eversion method by applying gentle pressure on left side of the abdomen to cause eversion of oviduct (vagina)^19^. Subsequently, 0.2 ml of the diluted semen was injected into the everted oviduct using tuberculin syringe to the depth of 2–4 cm into the cloacal orifice.

### Fertile eggs collection, storage and hatching out

Following 48 h after insemination, the fertile eggs were collected individually, cleaned and stored replicate-wise in cold storage room maintained at 65°F (physiological zero for chicken eggs is 68 °C, below which the embryonic development inside the egg stops) for five days. On the sixth day, the eggs were kept in room temperature for four hours and incubated in the setter (99.5°F and 55–60% relative humidity). The incubated eggs were examined for fertilization by performing the candling on 18th day of incubation and transferred to the hatcher (98.0°F and 70–80% relative humidity) and on the 22nd day chicks hatched out. The procedure of insemination, fertile egg collection, storage and hatching out has been carried out thrice (300 eggs) each time with 100 eggs per group to obtain consistent observations. Throughout this process, the replicate-wise identity of the eggs/chicks were maintained. The percent fertility and hatchability were determined using the following formula;

$$Fertility\,(\% ) = \frac{Total \,number\, of \,fertile \,eggs}{{Total\, number \,of \,eggs \,set\, in \,setter}} \times 100$$

$$Hatchability \,(\% ) = \frac{Total\, number\, of \,chicks \,hatched\, out}{{Total \,number\, of\, fertile \,eggs \,set\, in \,hatcher}} \times 100$$

Total of 82, 86, 93 and 88 chicks were hatched out from control, T1, T2 and T3 groups, respectively. All the hatched out chicks were vent sexed and group-wise 40 male chicks (N = 160 chicks) were selected and distributed in to four groups as per their maternal diet grouping. The chicks were randomly placed in five replicates consisting of eight chicks in each and reared in deep-litter system for 42 days. The chicks were fed with common broiler ration (pre-starter, starter and finisher) as ICAR recommendations under standard management practices including vaccination (Table 1).

**Table 1:** Ingredient and chemical composition of breeder and broiler diet.

Ingredients (%)	Breeder ration		Broiler ration		
	Male	Female	Pre-starter	Starter	Finisher
Corn	57.41	58.37	56.39	56.25	63.10
De-oiled rice bran	22.90	2.10	0.00	0.00	0.00
Rice bran oil	0.00	0.00	2.21	3.80	4.43
Soybean meal (47%)	15.60	28.90	37.50	36.20	30.21
Calcite	1.80	2.06	1.07	1.03	0.97
Oyster shell	0.00	6.20	0.00	0.00	0.00
Di-calcium phosphate	1.40	1.50	1.55	1.36	1.17
Trace minerals^a^	0.11	0.14	0.10	0.10	0.10
DL-Methionine	0.12	0.14	0.22	0.21	0.18
L-Lysine	0.13	0.06	0.37	0.24	0.0
Salt	0.26	0.26	0.35	0.34	0.34
Vitamin premix^b^	0.15	0.14	0.05	0.05	0.05
B-complex vitamins	0.15	0.01	0.025	0.025	0.025
Choline chloride	0.04	0.04	0.05	0.05	0.05
Toxin binder	0.04	0.04	0.10	0.10	0.10
Coccidiostat^c^	0.04	0.04	0.05	0.05	0.05
Nutrient composition (%)					
Crude protein	14.56	18.53	22.70	21.44	19.25
Metabolizable energy(kcal/kg)	2424	2670	3003	3102	3202
Calcium	1.08	3.52	0.98	0.98	0.95
Available Phosphorus*	0.40	0.42	0.45	0.42	0.40
Lysine*	0.69	0.89	1.35	1.28	1.14
Methionine*	0.33	0.39	0.62	0.58	0.56
Zinc (mg/kg)	40	40	40	40	40

*Calculated values.

^a^Mineral mixture contained manganese-91, zinc-91, iron-85, iodine-1.82, copper-30.24 and cobalt-0.365 mg.

^b^Vitamin premix contained vitamin A-16500 IU, B2-13 mg, D_3_-3200 IU and vitamin K-2 mg, thiamine-5 mg, pyridoxine-8 mg, Niacin-320 mg, cyanocobalamine-0.05 mg, vitamin E-95 mg, calcium D pantothenate-27.5 mg and folic acid-14 mg, calcium-30.1 mg.

^c^Coccidiostat supplied 125 mg of Di-nitro-ortho-toluamid.

### Body weight gain, feed consumption and efficiency

The chicks were individually wing banded and its body weight was recorded at hatch as well as at bi-weekly interval. Based on the body weight, the phase-wise body weight gain (g) was determined. The chicks had free access to pre-weight feed, water and the residual feed were measured bi-weekly to determine the phase-wise feed consumption (g/bird). The feed efficiency (g feed: g gain), was determined phase-wise and also overall.

### Intestinal development and absorptive surface area

The effect of maternal zinc supplementation on intestinal gross dimensions, histological development and absorptive surface area were assessed during the 14 and 42 days of feeding. The birds (10 per group) were anaesthetized with Ketamine + Xylazine (intra-muscular injection) and sacrificed by cervical dislocation method. The total intestinal tract as well as the segment-wise (duodenum, jejunum and ileum) length (cm/kg body weight) were measured.

The intestine segment samples were collected from representative birds (six) from each group. A 50-mm-long segment from the central part of the small intestine (jejunum) was collected and fixed in 10% neutral buffered formalin. The segments were sequentially processed for dehydration and obtained 4-μm-thick paraffin sections using microtome. The sections were stained using the hematoxylin and eosin (HE) stain. The cross-sectional tissue slides were evaluated under a Nikon eclipse Ci-S microscope coupled with a camera and the computer software NIS-D Version 4.0^20^ to determine the jejunal villi length, crypt depth and the villi length to crypt depth ratio. The jejunal absorptive area was determined by using the equation^21^.

$$M=\frac{\left(villus\, W\times villus\, L\right)+\left(villus\,\frac{w}{2}+crypt\,\frac{w}{2}\right)2-\left(villus\,\frac{w}{2}\right)2}{\left(villus\,\frac{w}{2}+crypt\,\frac{w}{2}\right)2}$$

M = intestinal absorptive surface area, villus W =  mean width of villi, villus L = mean length of villi, and crypt W = mean width of crypts.

### Statistical analysis

The data obtained on egg weight, feed intake, body weight gain, feed efficiency, intestinal histo-morphological parameters were subjected to analysis of variance (single factor) using the SPSS version 16.0. The observations obtained percentage (egg production, fertility and hatchability) were subjected to arcsine transformation before analysis. The means were compared to Tukey’s multiple range test for significance (*P* < 0.05).

## Results

### Breeders body weight, feed consumption, egg weight and production

**Fig. 1:**
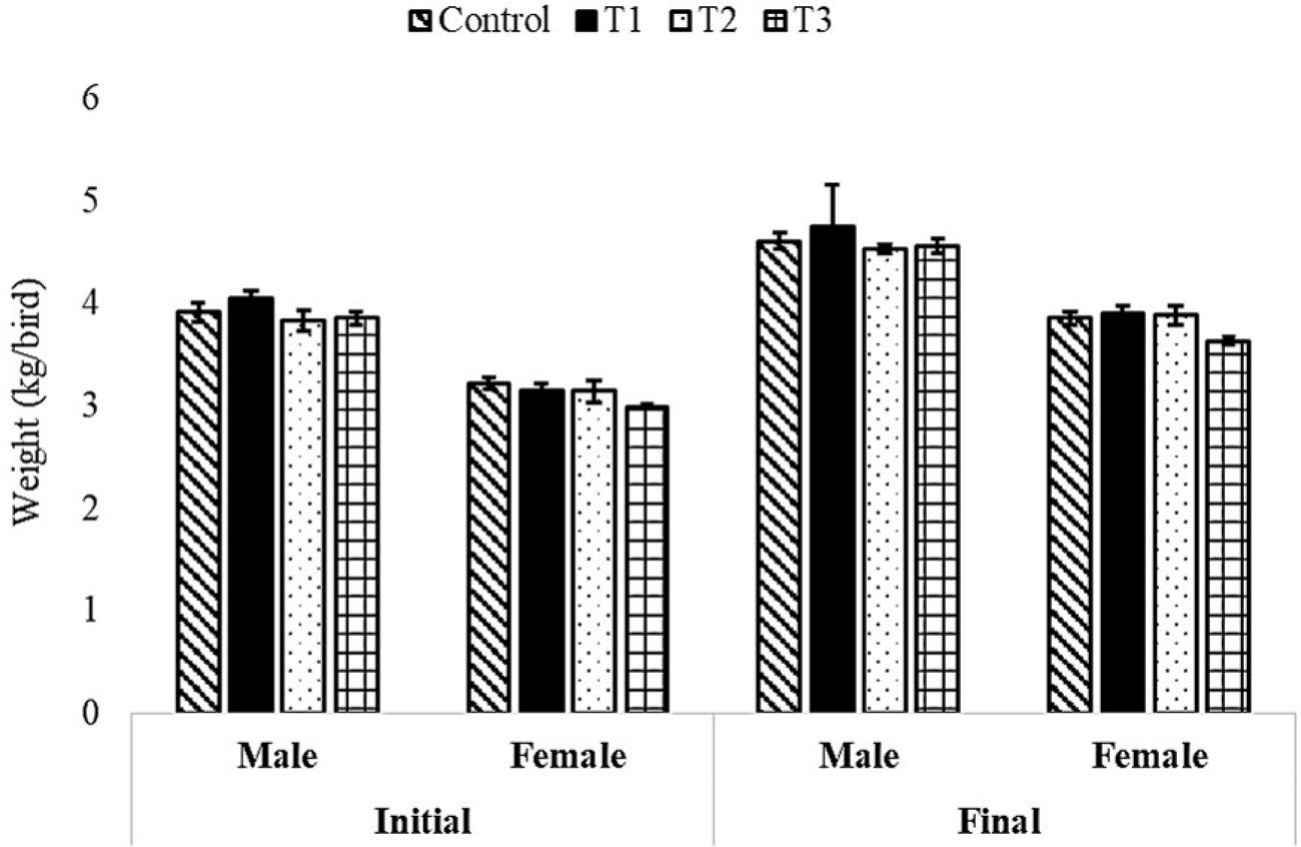
Effect of organic zinc (Zn-methionine) supplementation to the maternal diet on the body weight of male and female broiler breeder chickens.

**Fig. 2:**
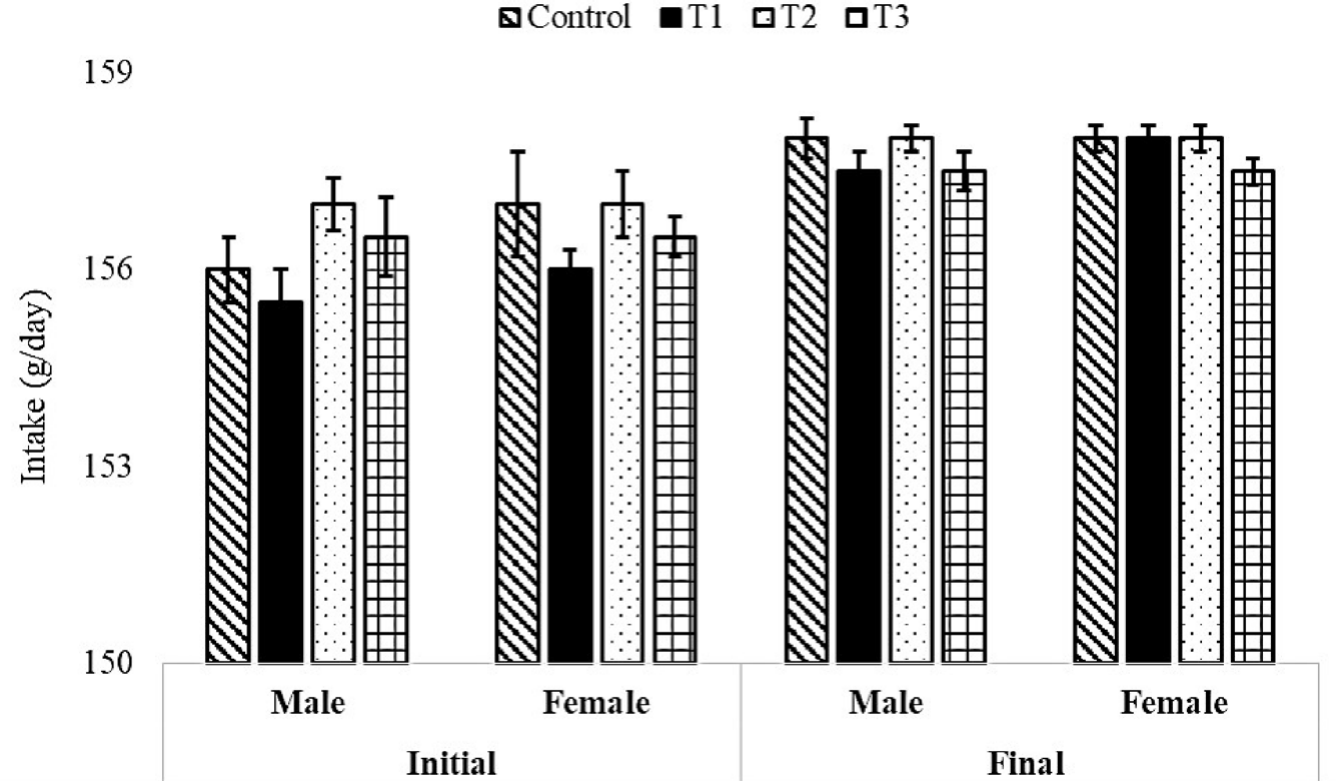
Effect of organic zinc (Zn-methionine) supplementation to the maternal diet on feed consumption in male and female broiler breeder chickens.

**Fig. 3:**
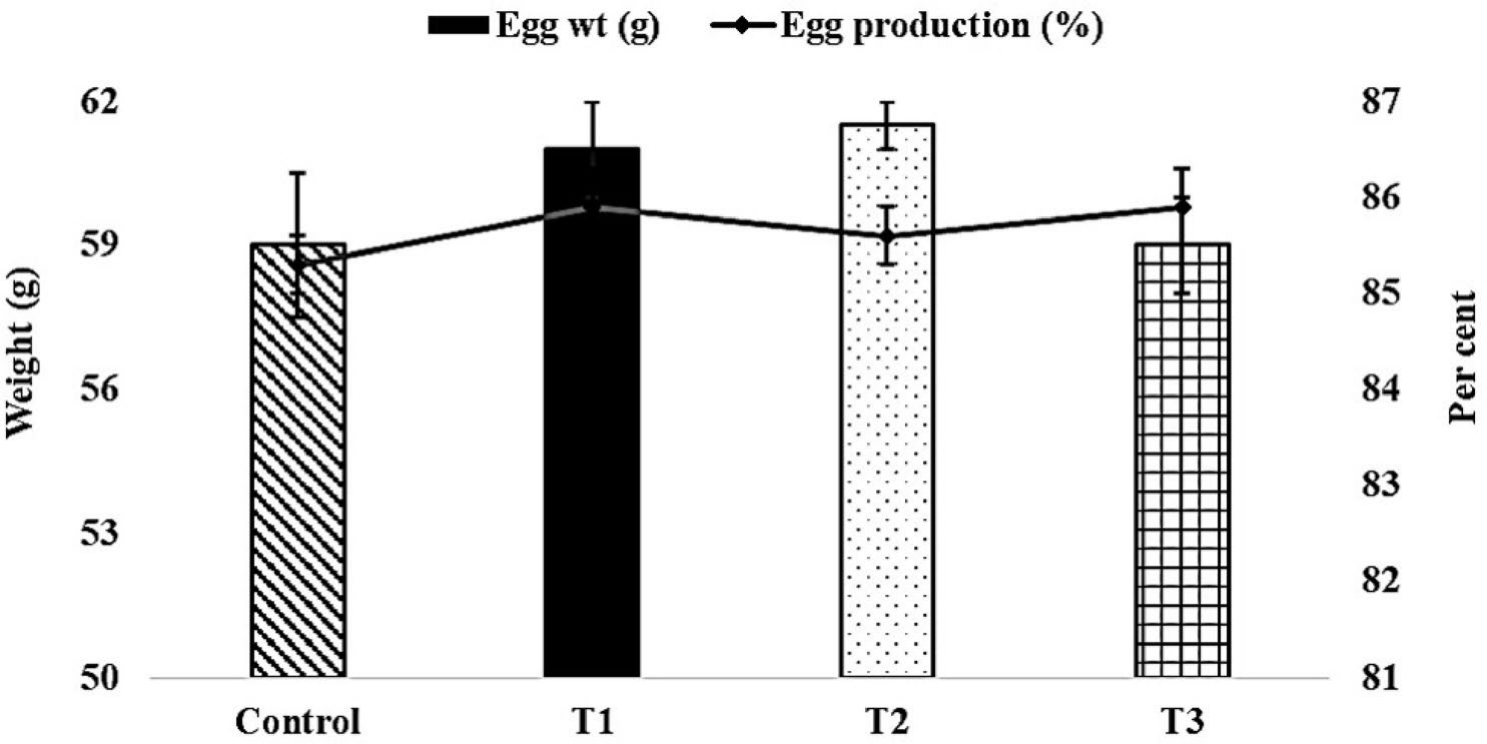
Effect of organic zinc (Zn-methionine) supplementation to the maternal diet on egg weight and egg production in female broiler breeder chickens.

**Fig. 4:**
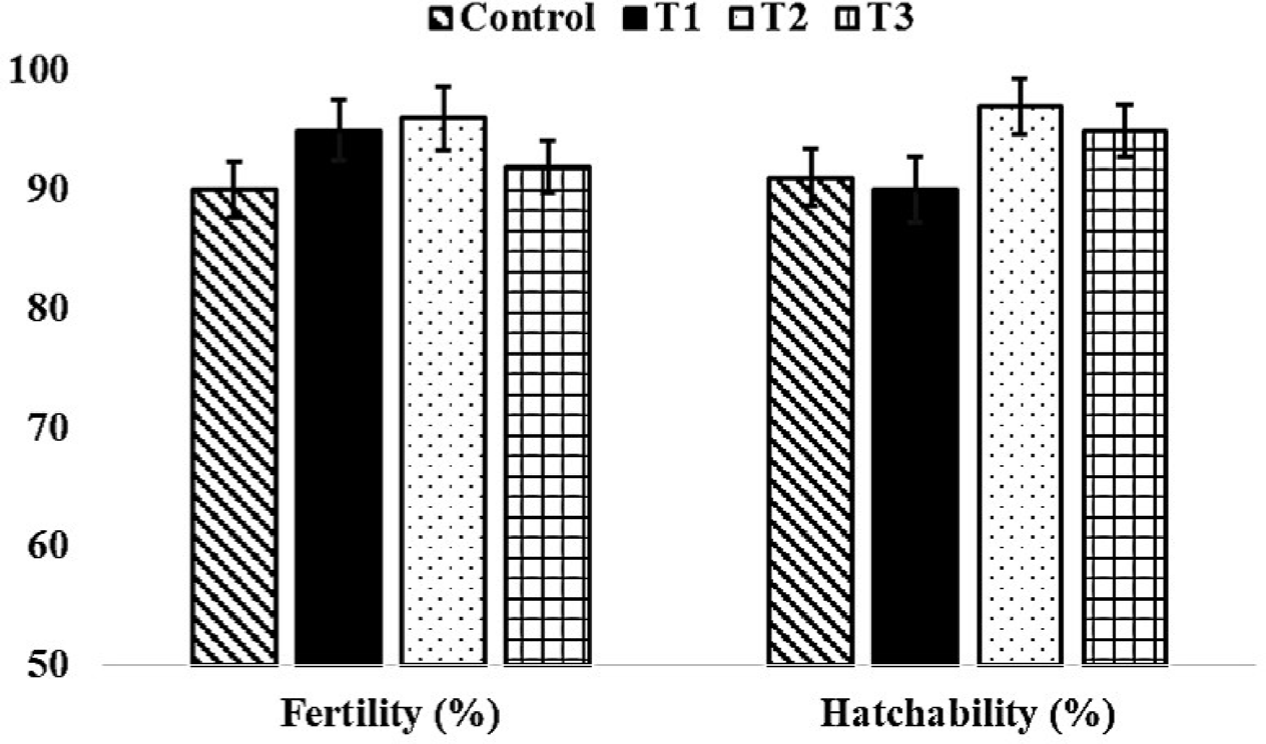
Effect of organic zinc (Zn-methionine) supplementation to the maternal diet on fertility and hatchability % in female broiler breeder chickens.

The initial and final body weight and feed consumption of both male and female broiler breeder birds were similar (*P* > 0.05) and not affected by the level of supplementary organic zinc (Figs. 1 and 2). Similarly, the egg weight and egg production were not affected (*P* > 0.05) by the graded level incorporation of zinc-methionine to the breeder’s diet (Fig. 3). The fertility and hatchability per cent on fertile eggs set basis were significantly (*P* < 0.05) higher in 60 mg organic zinc supplemented group compared to other levels and control groups (Fig. 4). Maternal supplementation of organic zinc (40 and 60 ppm) to broiler breeders basal diet had positive impact (*P* < 0.05) on the hatchability percentage. The additional supplementation of zinc could be more beneficial.

### Offspring’s growth performance

The maternal supplementation of zinc to broiler breeders diet, did not influence (*P* > 0.05) the body weight as well as the body weight gain of the broiler chicks up to six weeks of age. The feed intake (g) (1982.23 vs. 2214.70) and feed conversion efficiency (1.85 vs. 1.72) revealed no significant (*P* > 0.05) difference between the groups (Table 2).

**Table 2:** Effect of maternal supplementation of graded levels of Zn-methionine on the offspring’s body weight gain (g), feed consumption (g/bird) and feed efficiency (g feed/g gain) (Mean ± Standard Error) at different growth phases.

Attributes	Control	T1	T2	T3	*P* value
Body weight gain (g)					
Hatch weight	42 ± 1	41 ± 1	42 ± 1	41 ± 1	0.900
Second week	405 ± 7	413 ± 6	421 ± 7	453 ± 5	0.587
Fourth week	1242 ± 36	1280 ± 33	1346 ± 40	1375 ± 19	0.638
Sixth week	2067 ± 24	2127 ± 26	2210 ± 29	2268 ± 24	0.584
Feed consumption (g/bird)					
Second week	455 ± 11	460 ± 10	457 ± 10	492 ± 12	0.542
Fourth week	1109 ± 19	1147 ± 20	1224 ± 18	1231 ± 25	0.125
Sixth week	1482 ± 36	1591 ± 30	1587 ± 33	1645 ± 32	0.241
Total Feed Intake	3046 ± 18	3198 ± 21	3326 ± 24	3368 ± 25	0.367
Feed efficiency (g feed/g gain)					
Second week	1.25 ± 0.11	1.24 ± 0.13	1.21 ± 0.95	1.20 ± 0.11	0.213
Fourth week	1.33 ± 0.92	1.32 ± 0.14	1.32 ± 0.87	1.34 ± 0.99	0.375
Sixth week	1.80 ± 0.16	1.88 ± 0.14	1.83 ± 0.13	1.84 ± 0.12	0.321
Cumulative	1.56 ± 0.12	1.58 ± 0.86	1.56 ± 0.13	1.57 ± 0.25	0.463

Control: basal diet; T_1_: 20 mg/kg Zn; T_2:_ 40 mg/kg Zn; T_3_: 60 mg/kg Zn.

### Offspring’s intestine development, histo-morphology and absorptive surface

Intestinal morphological attributes (villi length, diameter and goblet cells) showed significant variation (*P* < 0.05) among the groups (Table 3). The maternal T3 (Zn-Me 60 mg/kg) recorded higher villi length (1106.63 µm) and diameter (853.82 µm) followed by T1 (954.34 µm), T2 (932.54 µm) and control group. Supplementation of zinc amino acid complex has higher villus length improved villus length without accompanying increase in crypt depth may indicate that there is less villous epithelial cell loss at the villus compared to supplementation with ZnSO_4_. Intestinal villi and crypt depth are important for digestive system. The ratio of villus height to crypt depth is important measures for evaluation of nutrient absorption. The intestinal absorptive surface area has been significantly higher in the supplemented groups compared to the control. This increase in absorptive surface area might be attributed to the increased villi length, width in the supplemented group.

**Table 3:** Effect of maternal supplementation of graded levels of Zn-methionine on the offspring’s intestine length, histo-morphological attributes and absorptive surface area (Mean ± Standard Error).

Group	Villi length (µm)		Villi Width (µm)		Crypt depth (µm)		Goblet cells (n/villus)		Intestinal length (cm)		Absorptive area (µm)	
	D14	D42	D14	D42	D14	D42	D14	D42	D14	D42	D14	D42
Control	613^c^ ± 12	1154^b^ ± 32	76^b^ ± 5	68 ± 15	139 ± 8	170 ± 11	133^ab^ ± 19	247 ± 21	85 ± 5	142 ± 4	52030^d^ ± 157	81585^d^ ± 341
T1	751^a^ ± 15	1386^a^ ± 43	111^ab^ ± 11	92 ± 12	107 ± 5	178 ± 17	88^b^ ± 11	158 ± 18	84 ± 3	146 ± 4	78882^b^ ± 197	84364^c^ ± 441
T2	695^b^ ± 14	1233^b^ ± 31	84^b^ ± 7	90 ± 18	109 ± 11	158 ± 27	129^ab^ ± 8	141 ± 29	88 ± 2	151 ± 5	95653^a^ ± 204	110078^b^ ± 378
T3	759^a^ ± 08	1407^a^ ± 19	138^a^ ± 18	83 ± 14	88 ± 9	131 ± 13	165^a^ ± 27	161 ± 24	92 ± 4	154 ± 3	65247^c^ ± 241	190255^a^ ± 504
*P* value	0.034	0.002	0.010	0.487	0.972	0.231	0.045	0.519	0.279	0.124	0.041	0.047

Control: basal diet; T_1_: 20 mg/kg Zn; T_2_: 40 mg/kg Zn; T_3_: 60 mg/kg Zn. ^abc^Means within column bearing different superscripts differ significantly (*P* < 0.05).

## Discussion

The role and requirement of major nutrients—energy and protein have been validated with reaching its limits^2^ but it is not the same with micro-nutrients which plays diverse functions. The trace mineral Zn is involved in more than 200 enzymatic reactions in the body and their metabolism could affect the overall performance. The study assessed the supplementary effect of organic Zn (Zn-methionine) on broiler breeder’s fertility, hatchability, post-hatch growth performance and intestinal development in offspring. The organic source was selected for the study due to their higher bioavailability and to avoid mineral–mineral interaction at the intestinal level rather than using the inorganic sources zinc oxide or zinc sulphate.

### Breeders body weight, feed consumption, egg weight and production

Supplementing organic Zn has improved the body weight gain in the broilers as they are in the actively growing birds which are under stress due Zn role in metabolism and anti-oxidant effect^22^. In the present study, the breeder birds were no more in their active growth phase (> 40 weeks of age) and maintained in ambient environment for more fertile eggs, no effect on body weight was observed. Zn could not impact body weight of the broiler birds that were maintained under ideal environment without deficiency^23^. The feed consumption of both male and female broiler breeder birds were similar (*P* > 0.05) as they were offered with restricted feed to maintain the body weight otherwise heavier birds have shorter laying sequence, larger eggs, reduced fertility and prone to vaginal prolapse^24^. Similarly, the egg weight and egg production were not affected (*P* > 0.05) by the graded level incorporation of zinc-methionine to the breeder’s diet. Studies indicated that the supplementation of Zn to breeder hens aged > 40 weeks had minimal effect on egg production but had significant increase in yolk Zn content^16^. The fertility and hatchability per cent on fertile eggs set basis were significantly (*P* < 0.05) higher in 60 mg organic zinc supplemented group. Maternal supplementation of either inorganic (ZnSO_4_) or organic (Zn-proteinate) to heat stressed broilers breeders resulted in higher hatchability due its anti-oxidant activity, maintenance of egg shell membrane and reproductive hormones^25,26^. About 40 mg/kg organic Zn could be ideal for higher fertility during early age (~ 33 weeks) and as the age of the birds increase an addition 20 mg (60 mg in total) was optimal for better fertility and hatchability in broiler breeders^27^. Considering the results of previous and current studies indicates that the additional supplementation of zinc could be more beneficial during the stress conditions.

### Offspring’s growth performance

The chick hatch weight were not influenced by the levels of organic Zn supplementation to the breeders as they were directly related to the egg weight which are similar among the groups. Sequential impact of egg weight on chick hatch weight is linear^28^, whereas the impact of hatch weight on market body weight or its prediction is dynamic due to feed, management, stress, etc.^29,30^. Contrast, the aged breeders (> 45 weeks) lays higher proportion of heavier eggs and hatches heavier chicks than younger flock (~ 35 weeks)^31^. The proportion of low quality broiler chicks need to be culled increases with age (45-wk)^32^ while the present study yielded good quality chicks across the groups indicating the ineffectiveness of the supplementary Zn. Considering the anti-oxidant role of Zn, the impact of organic Zn supplementation could be more during the summer/heat stress and aged flocks (> 45 weeks), where the birds experience metabolic/physiological stress and lays heavier eggs.

The maternal supplementation of Zn to broiler breeders diet, did not influence (*P* > 0.05) the body weight gain, feed consumption and efficiency of the offspring. Supplementing Zn either as inorganic (ZnSo_4_) or organic (Zn-glycinate) up to 80 mg/kg to the maternal diet did not affect the offspring’s growth performance. However, when the hatched out chicks were fed additional Zn (24 mg/kg basal diet + 80 mg ZnSo_4_) did improve the growth performance in chicks^15^. Supplementation of different sources Zn-methionine, ZnSO_4_, encapsulated Zn to the broiler ration (≥ 40 mg/kg) did not influence the growth performance^33–35^. In the present study, broiler breeders were fed with basal diet containing Zn 40 ppm and the offspring’s were fed with uniform broiler diet (pre-, starter and finisher) with standard zinc content (40 ppm). However, supplementation of zinc as sulphate/amino acid complex to the broilers fed with deficient/low zinc diet improved the body weight and feed efficiency^36–40^. Results highlights that the zinc supplementation will be beneficial only when the diets were insufficient (< 40 mg/kg) in Zn content.

### Offspring’s intestine development, histo-morphology and absorptive surface

The changes in the intestinal histo-morphological attributes such as villi length, diameter and absorptive surface area in the supplemented groups could be due to higher availability of Zn from yolk. Both the intestinal villi length and crypt depth are important for digestive system. An increased villus length is associated with increased nutrient digestion and absorption, increase in brush border enzymes and nutrient transport systems^41^. Organic zinc were more efficient in helpful of intestinal morphology with an increased intestinal villi length in different animals^42,43^. Zn is essential for the proliferation of cell and synthesis of protein at crypt base^44^. Supplementation of organic Zn (as amino acid complex, lactate) resulted in higher villus length and villi height to crypt depth ratio in different intestinal segments^35,39,45,46^. This increase in absorptive surface area might be attributed to the increased villi length, width in the supplemented group. The ratio of villus height to crypt depth is an important measure for evaluation of nutrient absorption. Increasing intestinal villus height has positive correlation with epithelial cell numbers^47^. Improved villus length without accompanying increase in crypt depth may indicate that there is less villous epithelial cell loss in birds compared to supplementation with ZnSO_4_. The observed effects on the intestinal histo-morphology might be due to additional Zn available to the chicks from the yolk of supplemented group birds. Maternal Zn supplementation increased the yolk Zn content (72 vs. 55 mg) but the content in the albumen was unaffected^48^. Zn in addition to its role as co-factor/co-enzymes for various enzymatic reactions, they also modulates cell signaling, protein kinase activation and intestinal cell proliferation, epithelial barrier functions through tight junctions^49,50^. The translation of these improvements to offspring’s performance were not observed in the study which might be due to combination of management conditions and sufficient Zn content in the basal diet.

## Conclusion

Maternal supplementation of organic Zn (Zn-methionine) to broiler breeders can be beneficial to have higher fertility and hatchability but doesn’t have trans-generational effects. For the study, the breeder birds were maintained under optimal management conditions and not exposed to any environmental stress, whilst supplementation could be beneficial during heat or metabolic stress to produce more fertile eggs and higher hatchability. The maternal supplementation could enhance the chicks performance when the offspring were fed with insufficient dietary Zn (< 40 mg/kg). The impact on intestinal histo-morphology could be beneficial in terms of performance when the offspring’s encounter biotic and abiotic challenges during their post-hatch growth phase.

## Data Availability

The data generated from the current study are available from the corresponding author on request.
